# Surface topography and contact mechanics of dry and wet human skin

**DOI:** 10.3762/bjnano.5.147

**Published:** 2014-08-22

**Authors:** Alexander E Kovalev, Kirstin Dening, Bo N J Persson, Stanislav N Gorb

**Affiliations:** 1 Department of Functional Morphology and Biomechanics, Zoological Institute, University of Kiel, Am Botanischen Garten 1-9, D-24098 Kiel, Germany; 2 IFF, FZ-Jülich, 52425 Jülich, Germany

**Keywords:** contact mechanics, interface fluid, roughness power spectrum, skin tribology

## Abstract

The surface topography of the human wrist skin is studied by using optical and atomic force microscopy (AFM) methods. By using these techniques the surface roughness power spectrum is obtained. The Persson contact mechanics theory is used to calculate the contact area for different magnifications, for the dry and wet skin. The measured friction coefficient between a glass ball and dry and wet skin can be explained assuming that a frictional shear stress σ_f_ ≈ 13 MPa and σ_f_ ≈ 5 MPa, respectively, act in the area of real contact during sliding. These frictional shear stresses are typical for sliding on surfaces of elastic bodies. The big increase in friction, which has been observed for glass sliding on wet skin as the skin dries up, can be explained as result of the increase in the contact area arising from the attraction of capillary bridges. Finally, we demonstrated that the real contact area can be properly defined only when a combination of both AFM and optical methods is used for power spectrum calculation.

## Introduction

The tribology of human skin is of great importance in sports, medicine, and cosmetics [1–2]. It is a rather complex topic due to the layered morphology and the viscoelastic–plastic nature of the human skin. A modern view about this topic is presented in [3].

The top-layer of the skin (*stratum corneum*, about 20 μm thick) has a Young’s modulus of *E* ≈ 1–3 GPa, which is similar to rubber in the glassy region. It is well know that the effective elastic modulus of *stratum corneum* may decrease by a factor of 100–1000 with increasing water content down to values of the order of *E* ≈ 5–10 MPa in the wet state, which is comparable to rubber in the rubbery region [3–4].

The tissues beneath *stratum corneum* are very soft. This has been demonstrated in indentation experiments on the inner forearm by using a macroscopic indentor (a ball with a diameter of about 1 cm). The measurements are explained well by using the Hertzian contact theory. The effective elastic modulus was found to be 10–40 kPa [3]. In a first approximation, a two layers model, with a thin stiff layer on top of a thick soft layer, is sufficient for a satisfactory description of the contact mechanics between the skin and the indentor.

The change of skin morphology and elastic modulus in the wet state contributes to the high friction of the wet human skin. However, the pattern of channels on the human skin surface, which is similar to the pattern on channels on the tree frogs toe pads [5], facilitates the fluid removal from the contact regions between skin and countersurface, increases the friction and enhances the grip between a fluid covered object and the human skin [3]. The strong reduction in the elastic modulus of the wet *stratum corneum*, e.g., due to sweating, results in a further increase of the contact area and of the friction, which may be crucially important in emergency situations.

In an earlier publication we have analyzed the frictional properties of skin by using the Persson contact mechanics theory. In [6] the surface topography of skin was measured by using an optical method with a resolution of the order of 1 μm. In this paper we report on AFM measurements at a higher resolution. From both optical and AFM data we have obtained the surface roughness power spectrum over all relevant length scales. This enabled us to perform a more accurate theoretical contact mechanics study of the frictional properties of skin, which we will report on in this paper.

## Experimental

**Figure 1:**
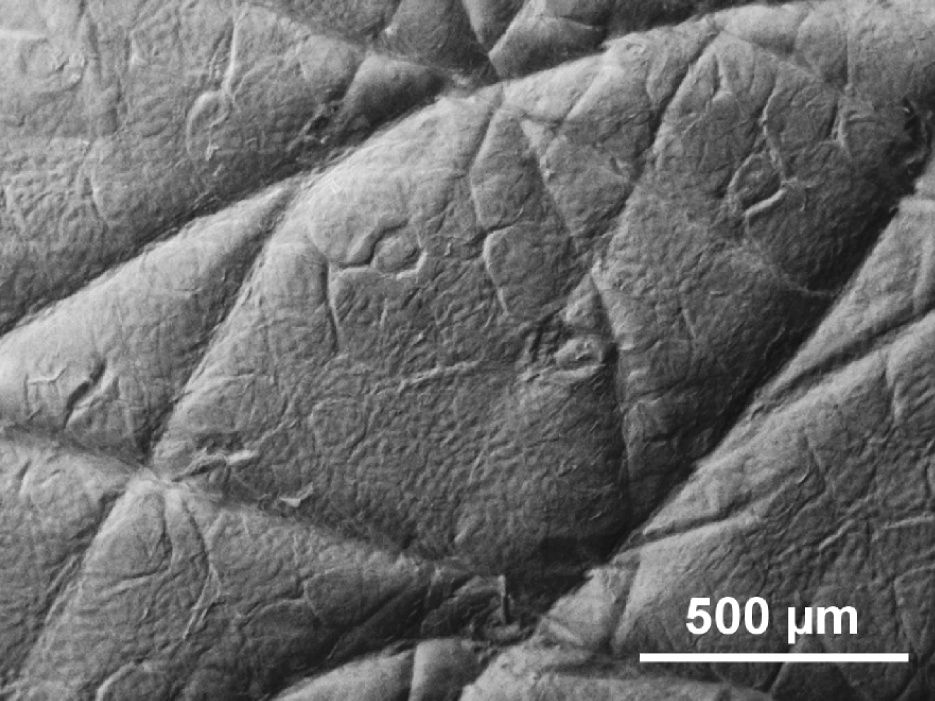
Scanning electron microscopy picture of a mould of wet human wrist skin.

**Figure 2:**
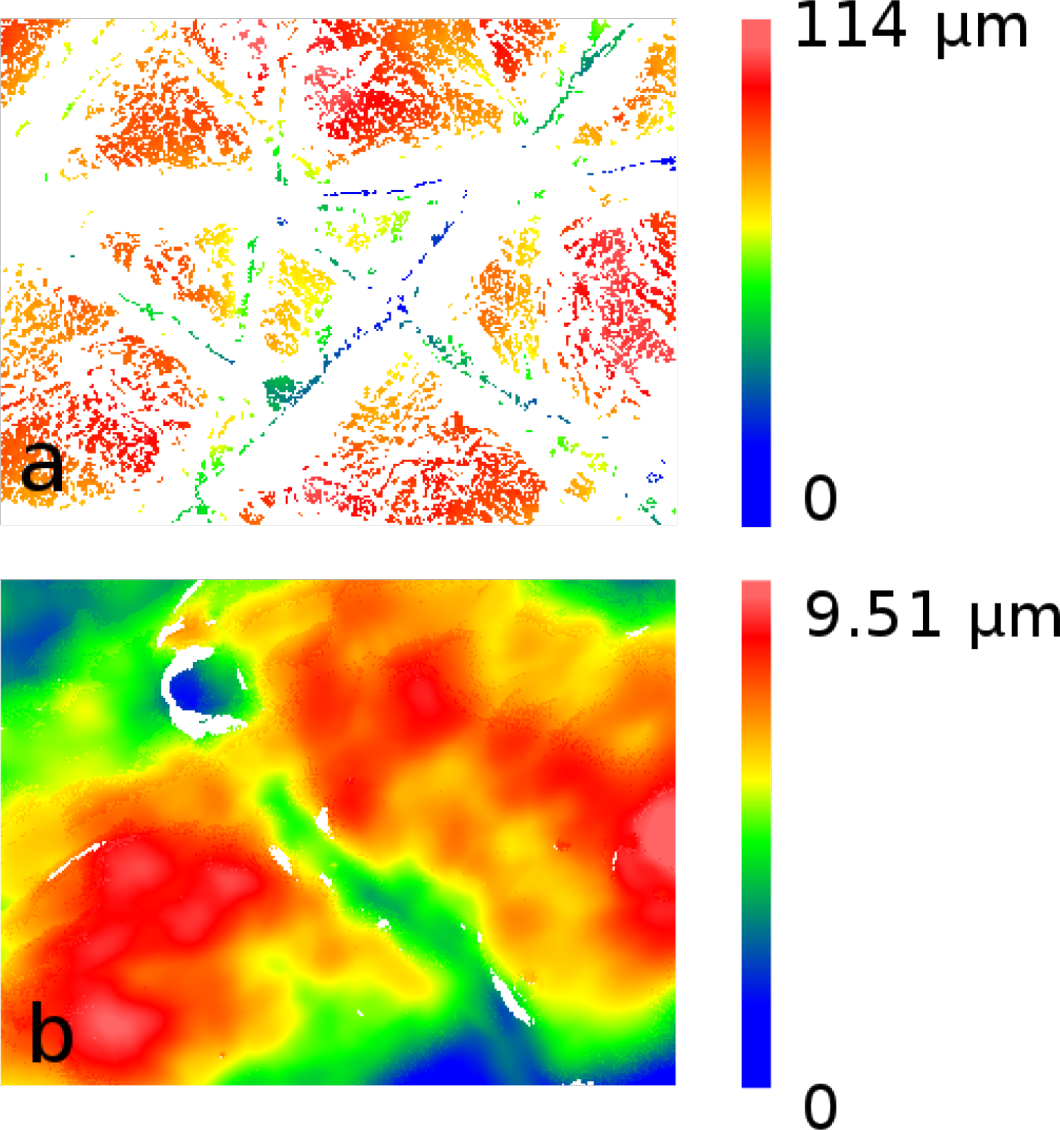
3D surface profiles of a mould of wet human wrist skin obtained by using white light interferometry at low (a) and high (b) resolution (only the measured raw data are presented). The image width is 1.4 mm in (a) and 140 μm in (b).

The topography of human wrist skin was analyzed in dry and wet states in the same way as described in [6]. The skin was washed with ethanol, wiped and dried for two minutes. To bring the skin in a wet state, a wet napkin was placed on the skin surface for 10 min. Afterwards, the skin was wiped and dried for two minutes. A two-component dental wax (President light body, Coltene, Switzerland) was used to prepare a negative mold of the skin. The negative mold was filled with Spurr’s low-viscosity resin and polymerized overnight at 70 °C. The images of positive epoxy replicas coated with gold–palladium (6 nm layer thickness) were obtained by using a Hitachi TM3000 tabletop electron microscope (Hitachi High-Technologies Corp., Tokyo, Japan) at an accelerating voltage of 3 kV (Figure 1). 3D surface profiles of the positive replicas were acquired by using a white light interferometer NewView 6k (Zygo, Middlefield, CT, USA) with 5× and 50× magnifications (Figure 2). The data obtained at low magnification are considerably discontinuous. Exact height could not be defined in white areas, Figure 2a. Besides, a speckle-like high-amplitude noise appear at low magnification. At high magnification the height may be properly defined almost in entire field of view, Figure 2b.

**Figure 3:**
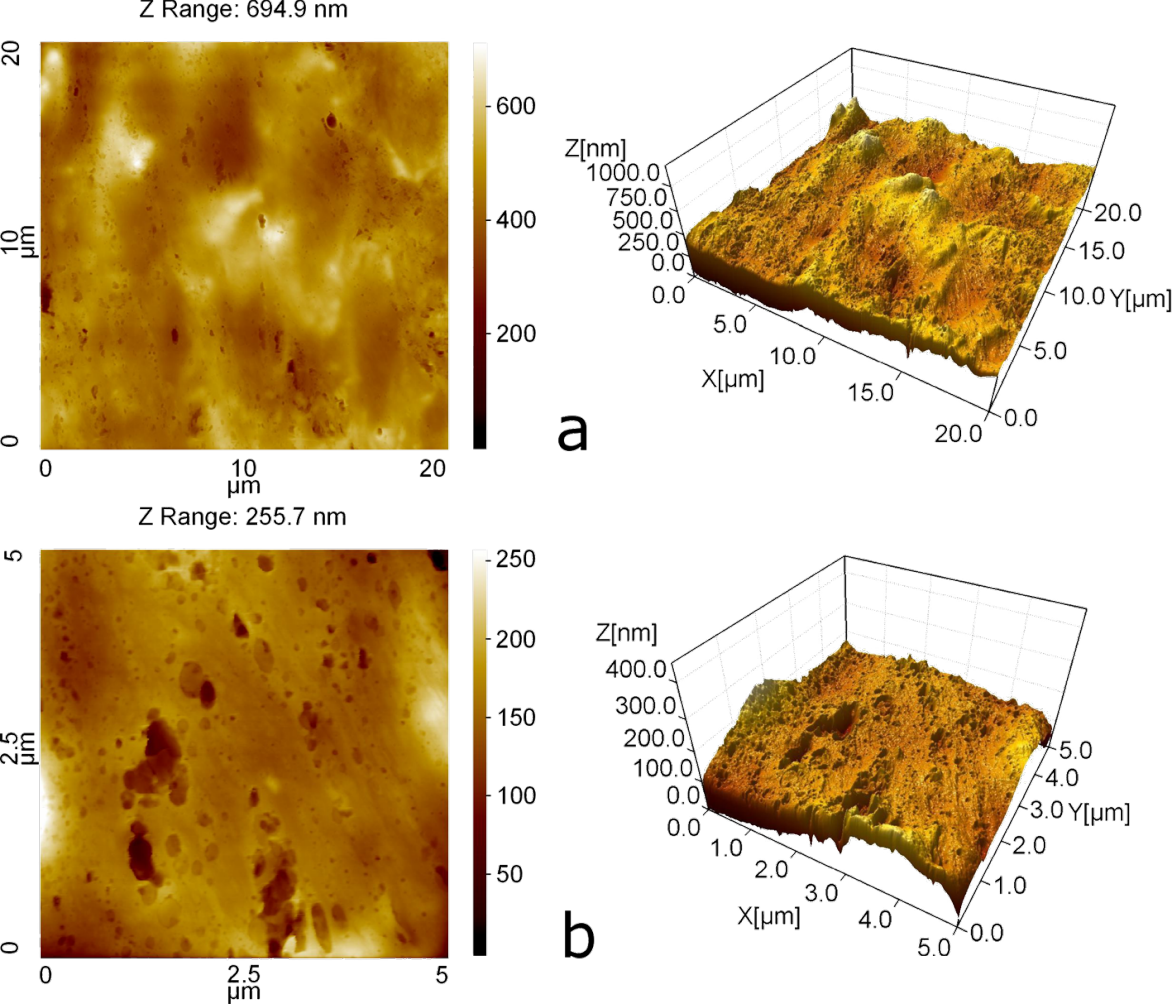
AFM images of a mould of wet human skin taken at (a) lower and (b) higher resolution.

For the AFM measurements a fragment of human wrist skin immediately after dissection was immersed in Ringer solution and placed on a Polysine™ slide (Gerhard Menzel GmbH, Braunschweig, Germany). The AFM measurements were performed with a NanoWizard^®^ AFM system (JPK Instruments AG, Berlin, Germany) equipped with a high density carbon tip (0.2 N/m, NanoWorld AG, Neuchâtel, Switzerland) in contact mode at 1024 × 1024 pixels resolution and 0.5 Hz scanning rate. Figure 3 was produced by using the software SPIP 5.1.2 (Image Metrology, Denmark).

Contact angles of water on skin were measured 5 times at different locations for each state of the skin by using a contact angle measurement device OCA20 (Dataphysics Instruments, Filderstadt, Germany). According to our measurements the advancing water contact angle on human wrist skin is (112.9 ± 1)° (mean ± s.e.m., *n* = 10) on dry skin and (121.4 ± 1.6)° (*n* = 9) on wet skin. The larger contact angle on wet skin could reflect either a change in the skin surface chemistry, or more likely may be due to the increase in the surface roughness of wet skin. The receding contact angle was not measured but it would be smaller than the advancing contact angle. In the literature, the values of the water contact angle on human skin range from 80 to 110° [7].

## Results and Discussion

### Surface roughness power spectrum of skin

Similar to [6] the measured height profiles *z* = *h*(**x**) were used to calculate the surface roughness power spectrum defined by [8–9]:

[1]
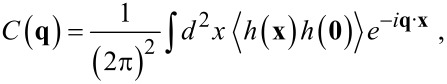


where **x** = (*x*,*y*) is the in-plane coordinate, <... > stands for the ensemble average, **q** = (*q_x_*,*q_y_*) is a two-dimensional wavevector of a particular cosines surface-roughness component with wavelength λ = 2π/*q* and orientation (in the *x*–*y*-plane) determined by the direction of **q**. *C*(**q**) depends only on |**q**| for surfaces with statistically isotropic roughness.

**Figure 4:**
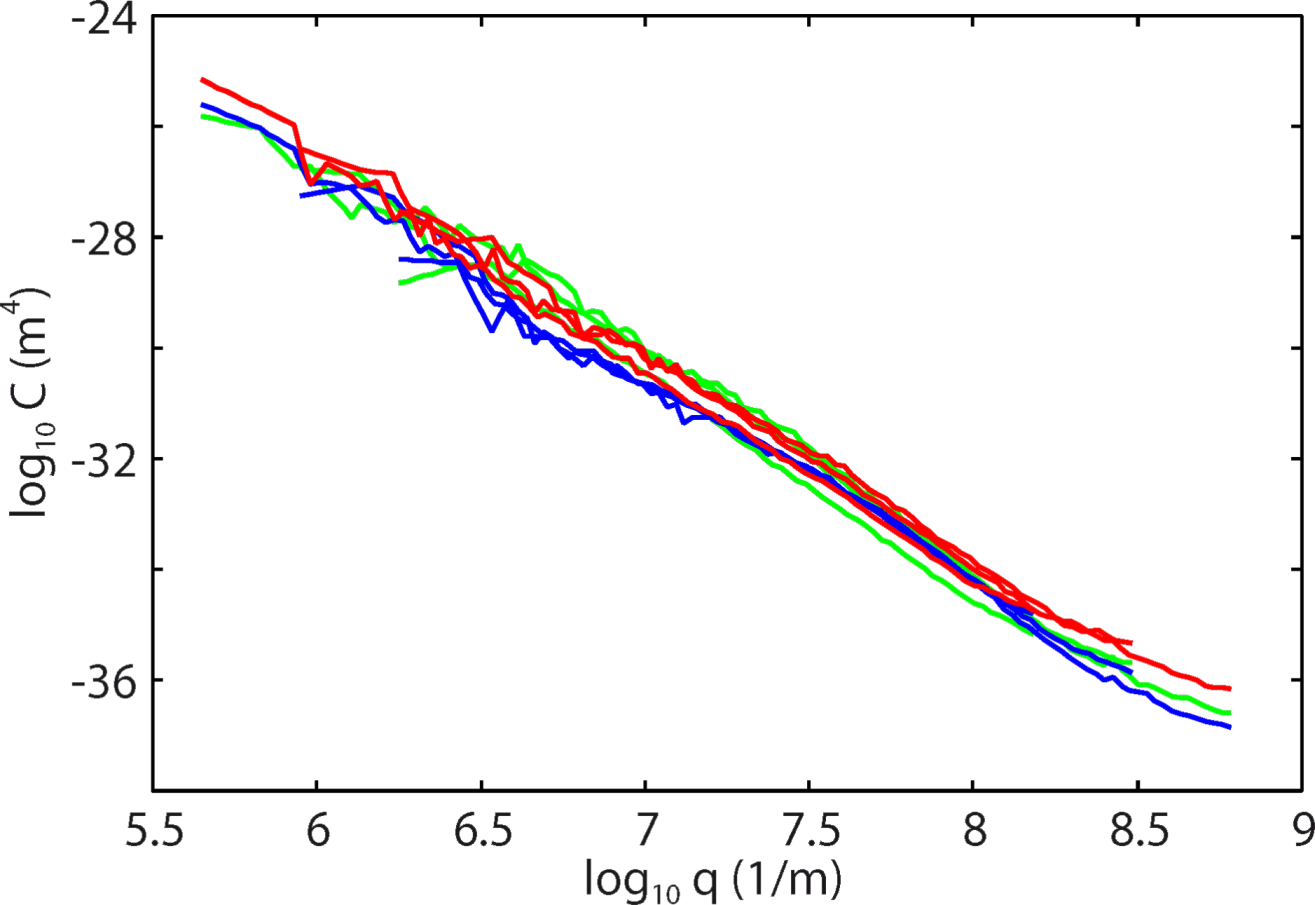
The power spectra as a function of the wave vector (log_10_–log_10_ scale), based on AFM topography data (green lines). The red lines are for dry skin, the blue lines for wet skin (calculated without AFM measurements).

Figure 4 shows the power spectra obtained from the AFM topography data, Figure 3, as a function of the wave vector (log_10_–log_10_ scale). The red and blue lines are calculated using the surface topography data from Figure 10 in [3] for dry and wet skin, respectively. The green lines in Figure 4 are calculated for wet skin with AFM data taken into account.

**Figure 5:**
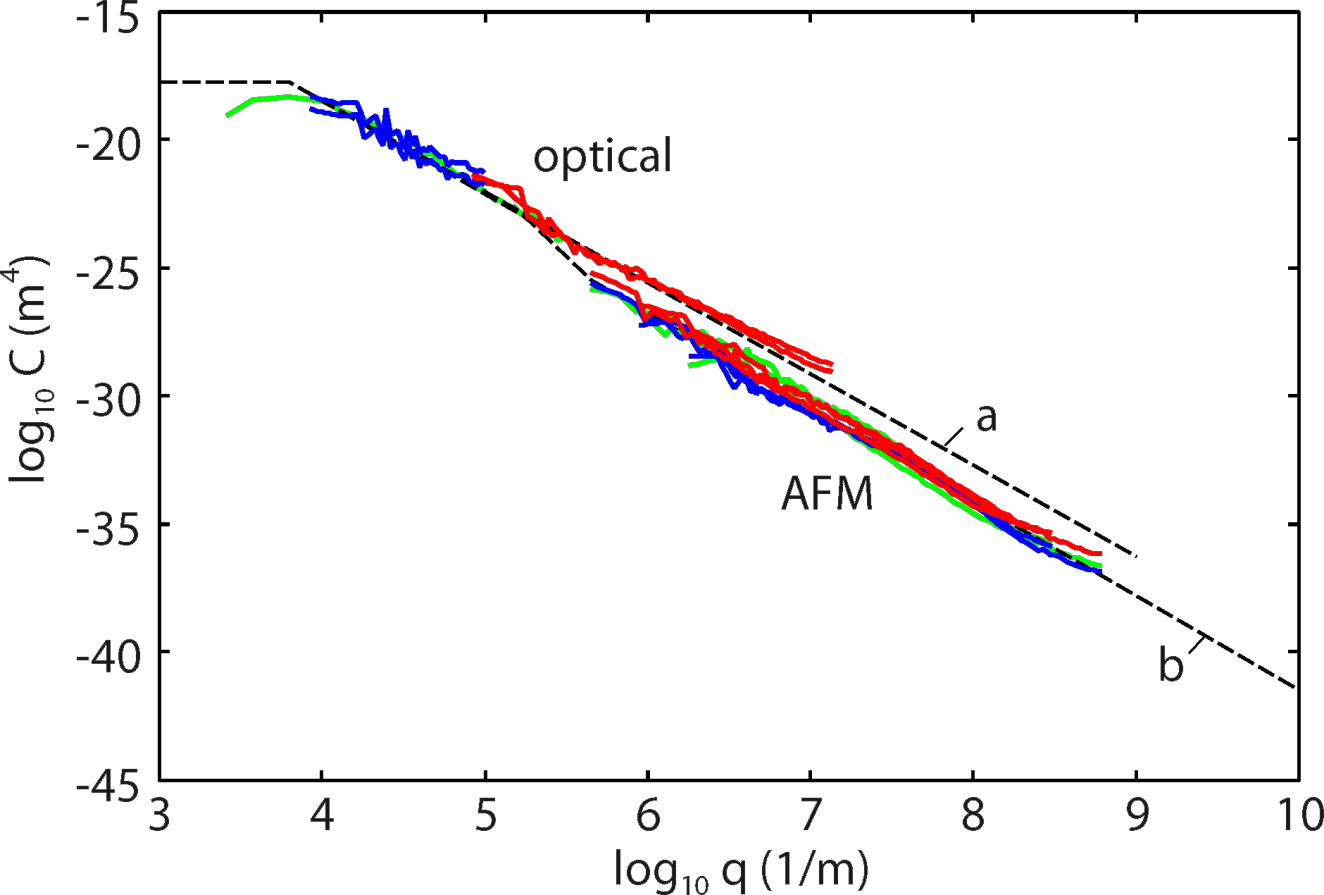
The power spectra as a function of the wave vector (log_10_–log_10_ scale). The dashed line denoted by **b** is the power spectra used in the calculations and correspond to a surface with the surface area *A*_tot_ = 1.3*A*_0_ and rms slope 0.9.

In Figure 5 we show the power spectra over a larger wavevector range including both the results from the white light interferometry [6] and the AFM measurements from Figure 4. The dashed line denoted by **b** is the power spectra used in the calculations presented below in Figure 9), and correspond to a self-affine fractal surface with a fractal dimension of *D*_f_ = 3 – *H* = 2.14 in the AFM region, a total surface area of *A*_tot_ = 1.3*A*_0_ (where *A*_0_ is the nominal or projected surface area) and an rms slope of 0.9. The smallest surface roughness wavevector is *q*_0_ = 10^3^ m^−1^ and the largest (cut-off) wavevector is *q*_1_ = 10^10^ m^−1^. The dashed line denoted **a** was used in the study presented in [6] and corresponds to a surface with the total surface area of *A*_tot_ = 2.7*A*_0_ and an rms slope of 2.8. Both power spectra correspond to surfaces with an rms roughness amplitude of ca. 22 μm.

### Contact mechanics of dry and wet skin

**Figure 6:**
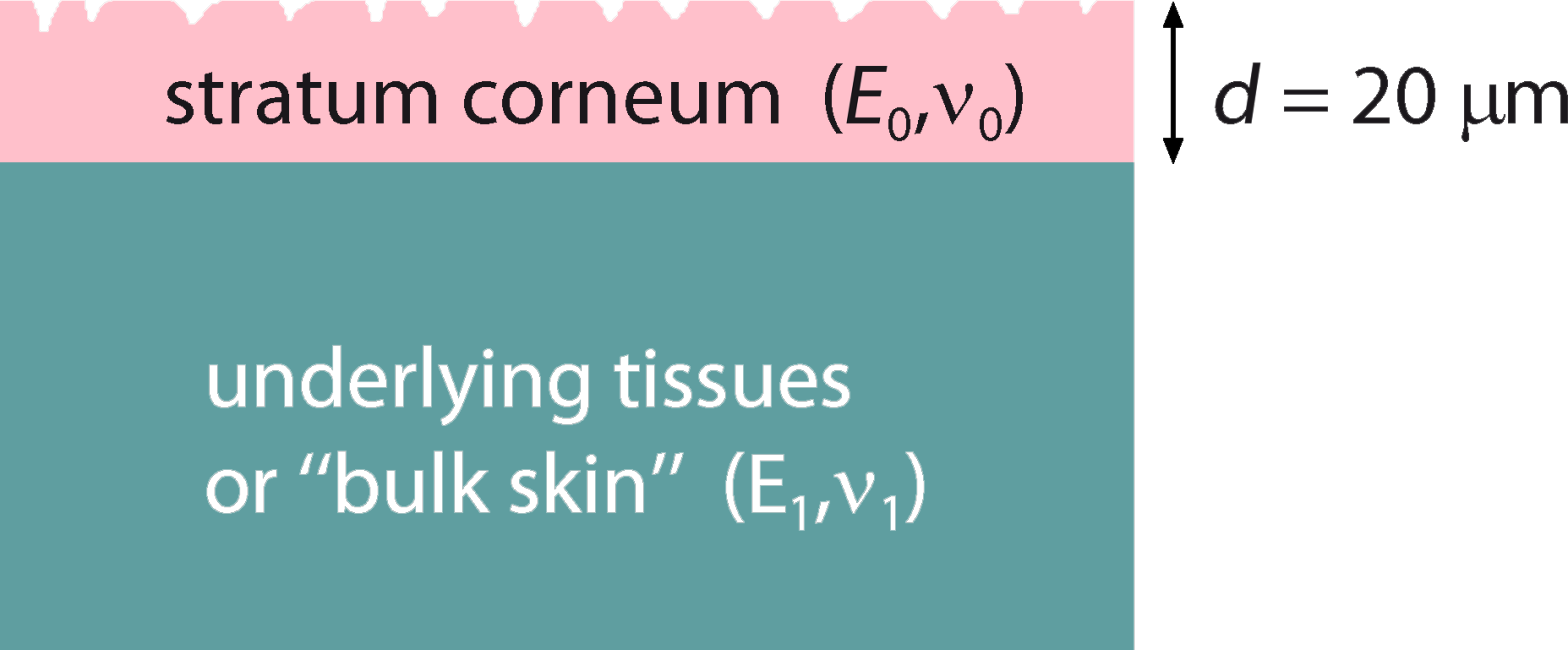
The model of the skin used in the calculations. The elastic modulus of the bulk skin is *E*_1_ = 20 kPa, and the Poisson ratio is ν_1_ = 0.5. The 20 μm thick top layer (*stratum corneum*) has a Young’s modulus of *E*_0_ = 7 MPa, in the wet state and of *E*_0_ = 1 GPa, in the dry state with a Poisson ratio of ν_0_ = 0.5. A penetration hardness of 50 MPa was assumed for dry skin.

The model of the skin used in the calculations is presented in Figure 6. The elastic modulus of the bulk skin is *E*_1_ = 20 kPa and the Poisson ratio is ν_1_ = 0.5. The 20 μm thick layer of *stratum corneum* has a Young’s modulus of *E*_0_ = 7 MPa in the wet state and of *E*_0_ = 1 GPa in the dry state with a Poisson ratio of ν_0_ = 0.5. Plastic deformation must be taken into account for the dry skin, because of the the high contact pressure. In the following calculations the plastic yield stress (or penetration hardness) of human skin was taken as σ_Y_ = 50 MPa [10], which is similar to the yield stress of most polymers, which are of the order of 100 MPa. We note that the yield stress of the skin is about 5% of its Young’s modulus, which is typical for many materials, e.g., for dry cellulose fibers. In fact, cellulose fibers exhibit elastoplastic properties very similar to the *stratum corneum*: Both absorb water strongly and swell by wetting, both have elastic moduli of the order of 10 MPa in the wet state and of the order of 1 GPa in the dry state [11]. The swelling (and elastic softening) of the skin in water take place while the water fills the multiple intracellular cisternae in *stratum corneum* [12]. The elastoplastic parameters given above are in agreement with experimental measurements [3,10], but a large spread of the parameter values is observed in different measurements.

We have used the Persson contact mechanics theory to analyze the contact between skin and a flat hard countersurface [6]. Shortly, when the interface is studied at a magnification ζ for which the surface roughness with wavevector *q* > *q*_0_ζ cannot be detected, the observed surface area is given by [9,13]

[2]
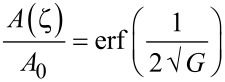


where erf(*x*) is the error function and

[3]
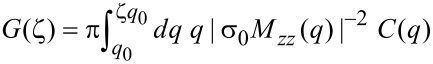


where σ_0_ = *p*_0_ is the applied stress or pressure. The linear response function *M_zz_* relates (in wavevector space) the surface displacement normal to the surface to the stress acting normal to the surface: *M_zz_*(*q*) = *u_z_*(*q*)/σ*_z_*(*q*). For a layered material of the type shown in Figure 6 it is given by 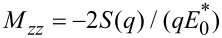, where 
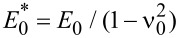
 and [14–16]

[4]
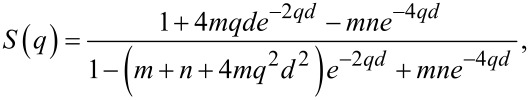


where


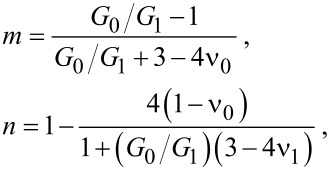


where *G*_0_ = *E*_0_/2(1+ν_0_) and *G*_1_ = *E*_1_/2(1+ν_1_) are the shear moduli for solid 0 and solid 1, respectively.

**Figure 7:**
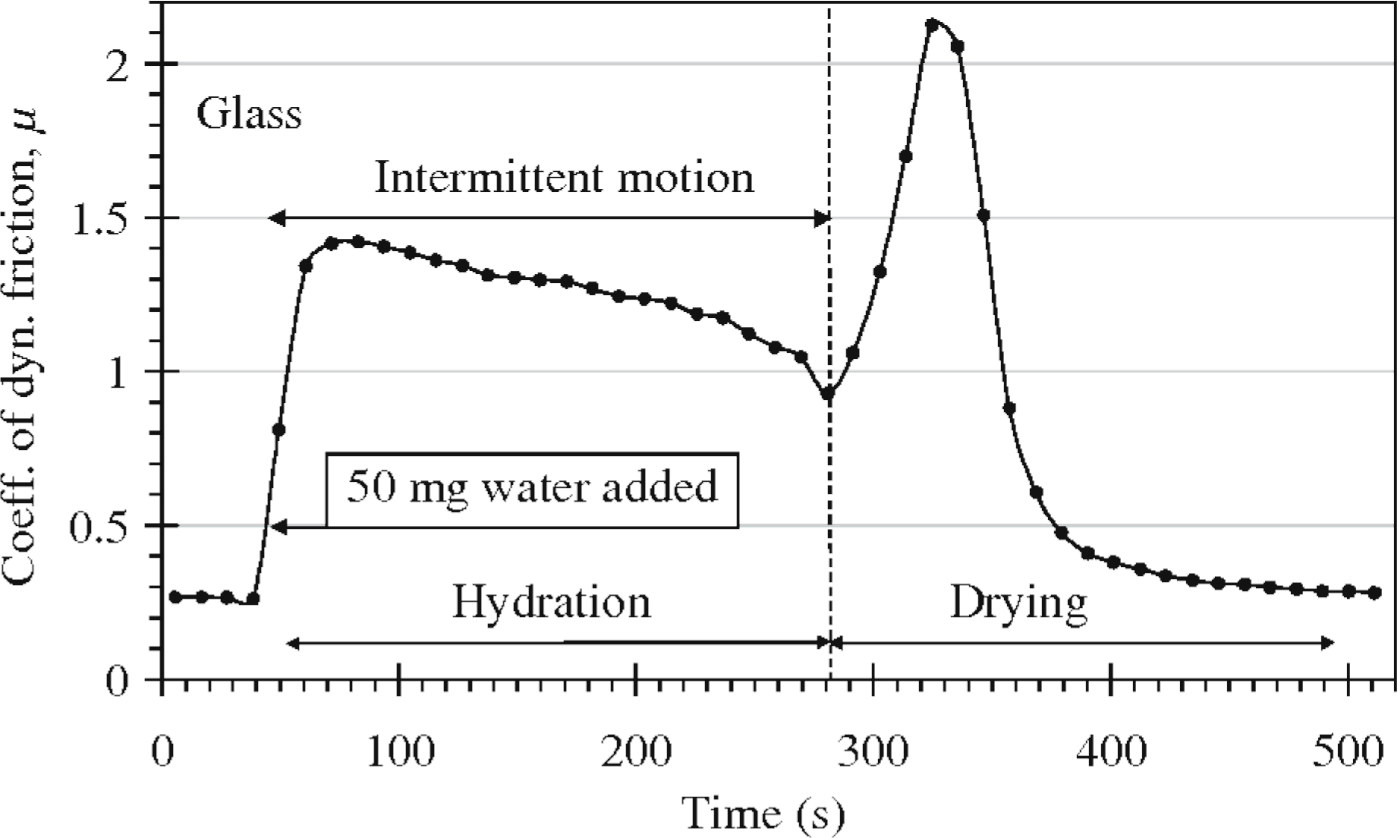
The friction coefficient of skin for a glass ball (*R* = 0.8 cm) at a sliding velocity of 0.8 cm/s and a normal load of 0.5 N during wetting/drying. Adopted from [3] with the permission of the authors.

**Figure 8:**
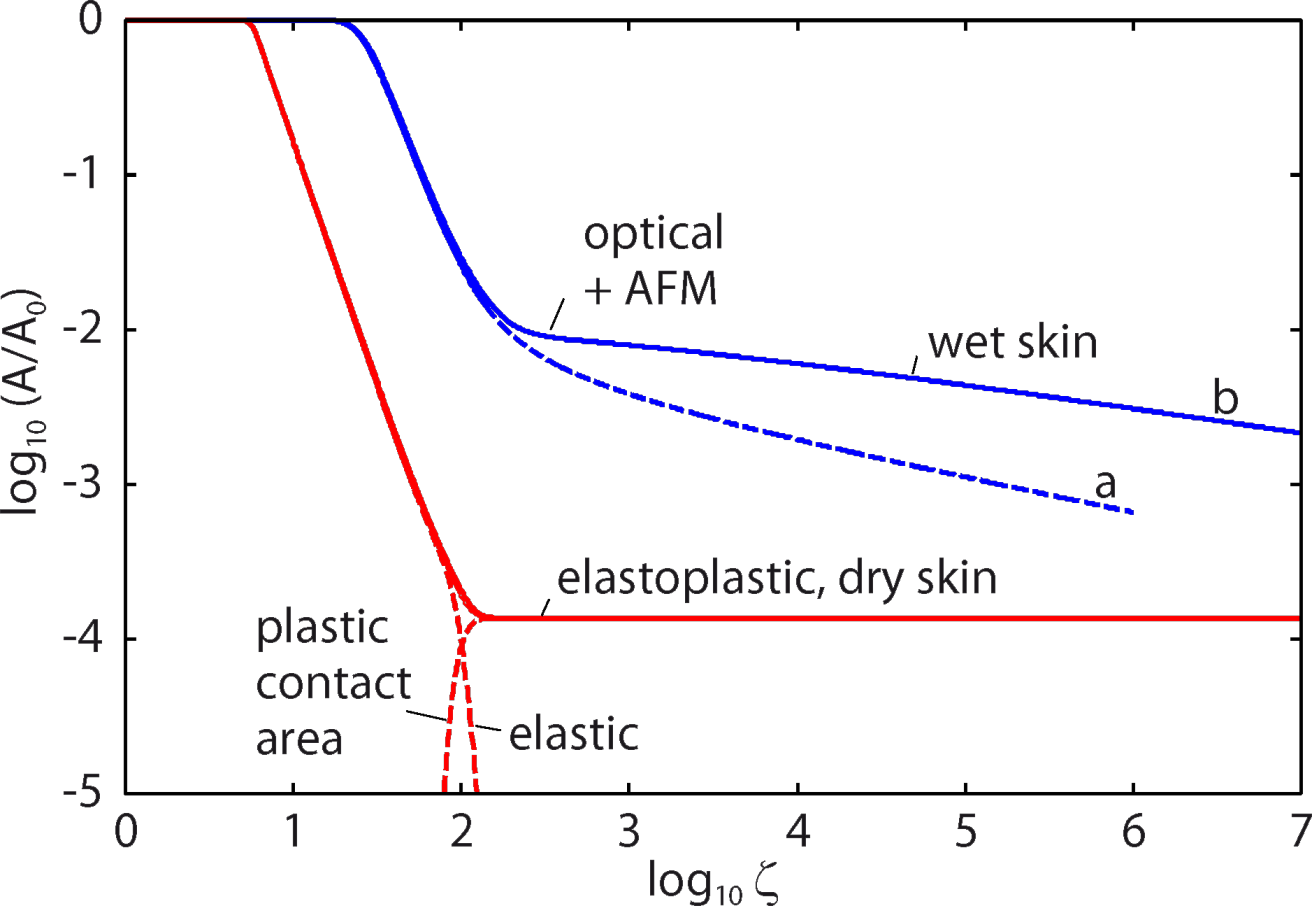
The ratio of the contact area *A* to the area of the nominal contact area *A*_0_ as a function of the lower scale magnification ζ included in the calculation in a log_10_–log_10_ scale. The blue and the red curves correspond to wet and dry skin. The solid and dashed blue curves use the surface power spectra denoted by **b** (optical and AFM data pooled together) and **a** (optical data only) in Figure 5, respectively. The skin model shown in Figure 6 is used in the calculations. The squeezing pressure was assumed as *F*_N_/*A*_0_ = *p*_0_ = 6.83 kPa.

In all the calculations presented below we have assumed a squeezing pressure *F*_N_/*A*_0_ = *p*_0_ = 6.83 kPa, which is the average nominal contact pressure in the experiments reported on in Figure 7. Figure 8 shows the area of contact (in units of the nominal contact area *A*_0_) as a function of the magnification ζ of the highest roughness components included in the calculation (log_10_–log_10_ scale). The blue and red curves in Figure 8 are for wet and dry skin, respectively. The solid and dashed blue curves use the surface power spectra denoted by **b** and **a** in Figure 5, respectively. For dry skin plastic yielding occurs already for small wavevector values (corresponding to long-wavelength roughness), for which the two power spectra with/without AFM data are identical. For this reason the contact mechanics for dry surfaces are the same using the two different power spectra. However, for the wet skin the power spectrum **a** result in a much smaller contact area than the power spectrum **b**. The reason is that the rms slope ξ (which is determined mainly by the large wavevector region of the power spectrum) of the surface in **a** is much larger than that in **b**, and the contact area is approximately proportional to 1/ξ.

The resolution of the instrument used to study surfaces determines the apparent contact area. Determination of the real contact area can be achieved only with an instrument having atomic resolution, such as AFM. Measurements based on the standard microscopy techniques, such as white light interferometry) have a resolution limited by the wavelength of the used light. Therefore, regions smaller than some fraction of the wavelength of light would appear as being in full contact and the contact area will be overestimated. At the highest magnification using the power spectra **b** the contact area is *A*/*A*_0_ = 2.15 × 10^−3^ for wet skin and 1.37 × 10^−4^ for dry skin. For dry skin complete plastic yielding occurs in all contact regions so that *A*/*A*_0_ = σ_N_/σ_Y_ = 1.37 × 10^−4^. Plastic deformation starts at *q* ≥ 10^5^ m^−1^ corresponding to a wavelength of λ ≤ 2π/*q* ≈ 60 μm. The values of the friction coefficients, μ ≈ 0.25 for dry skin and μ ≈ 1.4 for wet skin, could be explained by frictional shear stresses of about 13 MPa for the dry surface and of about 5 MPa for the wet surface. These values are very similar to the frictional shear stresses for sliding on polymers [17], or for many thin (ca. 1 nm) confined fluid layers between hard surfaces [18]. They are also similar to the frictional shear stresses in the area of contact for tread rubber sliding on different surfaces [19], during which for sliding velocities of the order of cm/s the frictional shear stress is typically of the order of 2–8 MPa.

### Capillary adhesion

In this section we evaluate different factors determining the tribological behaviour of wet skin, which is described in [3], following the line presented in [6]. The sliding friction of a glass ball was measured at a sliding velocity of 0.8 cm/s and a normal load of 0.5 N (Figure 7). At *t* ≈ 30 s, a water droplet, Δ*V* = 50 μL, was added to the sliding track. This corresponds to an average water film thickness on the track of the order of Δ*V*/*LD* ≈ 90 μm, where *L* ≈ 8 cm is the stroke length and *D* ≈ 0.8 cm the width of the (nominal) Hertzian contact region. A uniform water film of thickness Δ*d* = 90 μm evaporates in about 500 s at room temperature, 50% relative humidity, and an evaporation rate of 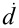 ≈ 1.7 × 10^−7^ m/s (see below). This is exactly the time period necessary for the friction to return to the dry state value (Figure 7). The sharp increase of the friction coefficient that appears as the water evaporates, Figure 7, might result from the increase in the area of real contact arising from the attractive force of capillary bridges. We can take into account capillary bridges in an approximate way as described in detail in [20]. That is, water is placed at the interface between the skin and the glass surface in all regions where the separation is less than the water layer thickness:

[5]
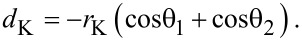


The negative Laplace pressure in the regions covered by water is described by

[6]
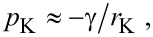


where γ is the surface tension of water. If Δ*A* is the surface area occupied by the capillary bridges then the attractive force is

[7]
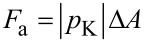


The contact area and the distribution of interfacial separations are determined by using the Persson contact mechanics model with the external pressure *p*_0_ replaced by the total pressure *p* = *p*_0_ + *p*_a_, where *p*_a_ = *F*_a_/*A*_0_. In this mean-field approximation the force from the non-uniform distribution of capillary bridges is replaced by a uniform pressure or stress *p*_a_. Note that to calculate Δ*A* one has to know the pressure *p* = *p*_0_ + *p*_a_. Since *p*_a_ depends on Δ*A* [9] there is an implicit equation for Δ*A* or *p*_a_, which can be solved, e.g., by iteration.

**Figure 9:**
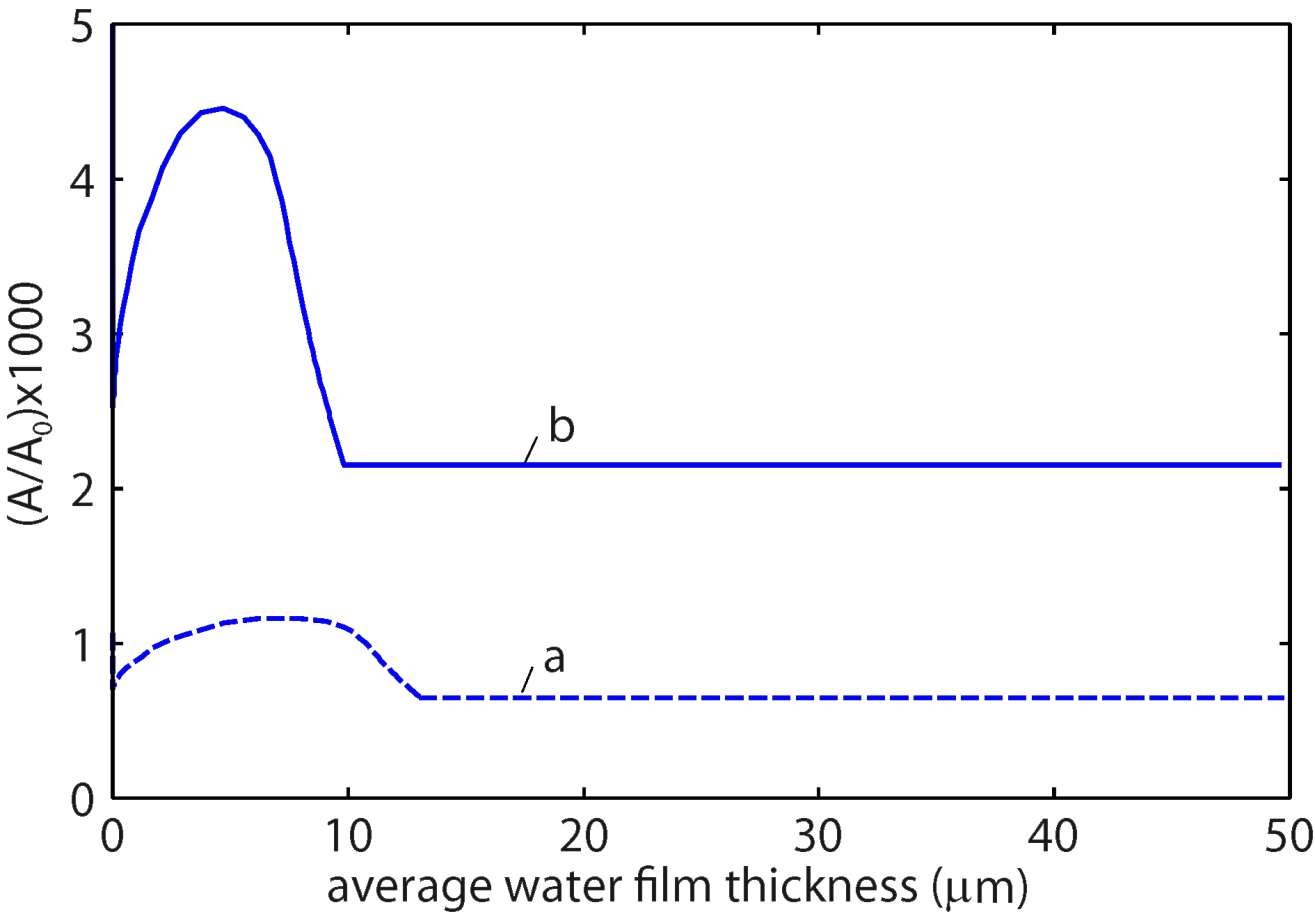
The area of real contact (in thousandths of the nominal contact area, *A*_0_) as a function of the average water film thickness *d*. For *d* > 12 μm water fills the whole interface. The solid and dashed curves use the surface power spectra denoted by **b** and **a** in Figure 5, respectively. In the calculations we have used the skin model shown in Figure 6 and assumed a contact angle of 0° for water on glass and of 80° for water on skin. The squeezing pressure is *p*_0_ = *F*_N_/*A*_0_ = 6.83 kPa.

The area of real contact in thousandths of *A*_0_ is shown in Figure 9 as a function of the average water film thickness *d*. The solid and dashed curves use the surface power spectra denoted by **b** and **a** in Figure 5, respectively. Note that the surface with the smaller rms slope (corresponding to the power spectra **b**) results in the largest contact area. For *d* > 12 μm the water covers all the interface (flooded condition). We have assumed a contact angle of 0° for water on glass and of 80° for water on skin. The increase in the contact area for an average water film thickness between 0 and about 10 μm is due to the formation of capillary bridges. Since the contact area is small compared to the nominal contact area, the area of real contact is proportional to the effective squeezing force *p*_0_ + *p*_a_. Therefore, at the point where the contact area is maximal, *p*_a_ ≈ *p*_0_, i.e., the attractive capillary pressure is of similar magnitude as the (nominally) applied pressure *p*_0_ ≈ 7 kPa. The width (in seconds) of the friction peak observed in Figure 7 corresponds to a change in the average water film thickness (due to evaporation) of ca. 10 μm, which is in beautiful agreement with the width of our predicted friction peak.

The water evaporation rate 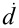 (change per unit time of the fluid film thickness *d*) is given by the empirical formula [21]

[8]
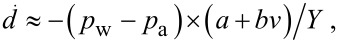


where *p*_w_ is the water saturation vapor pressure at the water (surface) temperature, *p*_a_ is the water vapor pressure in the air (which is the product of the relative humidity and the saturation water pressure at the air temperature and air pressure), and *v* the velocity of the air (some distance from the water surface) over the water surface. *Y* is the latent heat of evaporation (for water *Y* ≈ 2272 kJ/kg) and the coefficients *a* and *b* are *a* = 8.9 × 10^−5^ m^4^/(kg·s) and *b* = 7.8 × 10^−5^ m^3^/kg, respectively. If the water on the skin has a temperature close to the body temperature, say *T* = 35 °C, then *p*_w_ = 5.6 kPa. The experiments were performed at room temperature (*T* = 20 °C) and a relative humidity of 50% giving *p*_a_ = 0.5 × 2.3 kPa = 1.15 kPa. Hence, since *v* << 1 m/s, we get 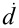 ≈ 1.7 × 10^−7^ m/s. Thus it takes Δ*t* = Δ*d*/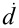 ≈ 60 s for the water film thickness to decrease by Δ*d* ≈ 10 μm, which according to the theory is the film thickness range over which the capillary attraction between the surfaces is effective. This is in beautiful agreement with the experimental data (see Figure 7).

We have assumed that the thermodynamic water–skin contact angle is θ_1_ ≈ 80°, and the water–glass contact angle θ_2_ ≈ 0°. Thus cosθ_1_ + cosθ_2_ ≈ 1 > 0 so that the interface is hydrophilic and attractive capillary bridges can form. In a second friction experiment Adams et al. [3] used a polypropylene sphere and in this case no increase in the friction was observed during drying. This is consistent with the fact that the water–polypropylene contact angle θ_2_ ≈ 102° so that cosθ_1_ + cosθ_2_ ≈ −0.03 < 0. Hence, the interface may be slightly hydrophobic resulting in a negligible interaction between the counterpart bodies during drying. For more strongly hydrophobic interfaces, e.g., skin in contact with Teflon in water, a dewetting transition may occur resulting in a dry contact area and an effective attraction between the skin and the countersurface [22].

## Conclusion

We studied the contact mechanics and friction for dry and water-lubricated human skin. The surface topography is studied by using two different methods, white light interferometry and AFM, which in combination allowed us to obtain the complete surface roughness power spectrum. The Persson contact mechanics theory is used to calculate the dependency of the apparent contact area on the magnification for both dry and wet skin. It was shown, that the proper estimation of the real contact area and attraction force is possible only if both types of surface profile measurements, AFM and white light interferometry, are used for the calculation of the roughness power spectrum. For dry skin plastic yielding becomes important and will determine the area of real contact at the highest magnification. The measured friction coefficient on both dry and wet skin can be explained assuming that a frictional shear stress σ_f_ ≈ 10 MPa acts in the area of real contact during sliding. This frictional shear stress is typical for sliding on elastomeric surfaces, and for nanometer thick confined fluid films. The big increase in friction, which has been observed for glass sliding on wet skin as the skin dries up, can be explained by the increase in the contact area arising from the attraction of capillary bridges. This effect is predicted to operate as long as the water layer is thinner than ca. 10 μm.
